# Multi-method ADHD diagnostics in children: CBCL and TRF lead the way

**DOI:** 10.3389/fpsyt.2025.1668149

**Published:** 2025-11-17

**Authors:** Luisa Himmelmeier, Robert Waltereit, Katja Werheid

**Affiliations:** 1 Clinical Neuropsychology and Psychotherapy, Faculty of Psychology and Sports Science, Bielefeld University, Bielefeld, Germany; 2 Department of Child and Adolescent Psychiatry, Psychotherapy and Psychosomatics, LWL-Hospital Marsberg, LWL-Klinik Marsberg, Marsberg, Germany; 3 Department of Psychiatry, Psychotherapy and Preventive Medicine, Ruhr University Bochum, Bochum, Germany

**Keywords:** attention-deficit/hyperactivity disorder, children, psychometric measurements, diagnostic accuracy, ROC

## Abstract

**Objective:**

Diagnosing ADHD in children requires multi-method examinations, yet the efficacy of this approach remains inconsistent. Our case control study investigated which standardized assessment methods most accurately predict an ICD-10 ADHD diagnosis in mixed settings (inpatient, outpatient, school).

**Methods:**

We examined 125 children aged 6–13 years, thereof 56 with an ADHD diagnosis (*M* = 9.7, *SD* = 2.09) and 69 without (*M* = 9.04, *SD* = 2.05). Our assessment included a children’s self-report questionnaire (Youth Self Report 11-18R, YSR), which was exploratorily used for younger children, and two objective tests, a Gameboy-administered Go/No-Go-task (QIKtest, 1) and a PC-administered continuous performance test (CPT, 2). Parents were asked to complete some questions on the child’s possible diagnoses and medication, and a parent questionnaire (Child Behavior Checklist 6-18R, CBCL). Teachers received the same questionnaire in adapted form (Teacher’s Report Form 6-18R, TRF). Classification accuracy was determined using receiver operating characteristic (ROC) analyses (Sensitivity, Specificity, Area under the Curve and Diagnostic Odds Ratio). A stepwise combination of indices was used to explore a multi-method procedure and its diagnostic accuracy.

**Results:**

CBCL and TRF achieved the highest classification accuracy focusing on inattention, followed by the YSR. Omission errors of CPT and QIKtest showed moderate classification performance while commission errors achieved the lowest. Combining CBCL, TRF and YSR showed superior diagnostic accuracy.

**Conclusions:**

Our results emphasize the relevance of multi-perspective questionnaire procedures for ADHD diagnosis despite potential acquisition challenges in clinical practice. Future research should develop more accurate objective test procedures and norm-based scales for children’s self-reports.

## 1 Introduction

Effects of treatment interventions in ADHD are assessed with heterogenous measurement methods, impeding the comparability of study results. Clinical judgment should be based on examination of the ADHD criteria according to clinical guidelines [e.g., (3)] and standard classification systems. However, deciding whether these criteria are fulfilled is difficult in many cases, and there is so far no gold standard for ADHD diagnostics (4–7). Use of multiple instruments (multimethod diagnosis) is therefore recommended (7–9), and a variety of measurement instruments is employed to quantify ADHD symptoms in children. In addition to the patient’s medical history (family, life circumstances, biography), the most common measures are parent, teacher, and children’s self-report questionnaires as well as continuous performance tests (CPTs).

Parent reports of children’s ADHD symptoms have been investigated by several studies in recent years, yet yielding inconsistent results. Some studies have found parent reports to be a positive predictor of an ADHD diagnosis (8, 10), which was explained by parents’ more extensive array of information (11). Other studies did not find an advantage of parent reports compared to teacher reports (12), arguing that parents are, like teachers, only present in a part of children’s life contexts, or might lack comparison with other children of the same age, or aim for amplified support for their children [e.g. (13–15)].

Teachers can evaluate children’s behaviors, interactions with peers and ability to adapt in contexts other than the home, and provide insights into group attitudes, social interactions, and performance behavior through the school context [e.g., (16)]. The majority of studies demonstrate lower diagnostic accuracy for teacher reports due to overestimation or underestimation of behavioral problems (17). In the domain of overestimation, the predominant reasons cited were reduced selectivity in teachers’ assessments of disruptive behavior in pupils with ADHD, opposed to relative immaturity (14), especially for children who are too young for their grade level or of male gender (18). As posited by Wienen et al. (16), overestimating ADHD symptoms would result in several advantages for teachers. These advantages include providing clear reasons for declining performance, facilitating working relationships with parents, and enhancing support to integration of children with behavioral problems in school contexts.

Conversely, underestimating ADHD symptoms has been attributed to anticipation of their possible consequences, influenced by the concern that children would face disadvantages associated with stigmatization (19). As indicated by Wienen et al. (16), this could be exacerbated by a critical attitude towards classifying nonconforming behavior as mental illness. Moreover, obtaining teacher reports is resource-demanding for all participants of the diagnostic process, and might expand the diagnostic process and the possible start of treatment (12).

Likewise, research on children’s self-reports has yielded divided evidence. Some studies reported that children with ADHD were more aware of their symptoms than previously assumed (20, 21) and can increase children’s motivation for treatments (19). Also, including children’s self-reports is in line with the principle of patient-centered care (22). On the other hand, several studies have identified a higher prevalence of response biases in children with ADHD compared to those without ADHD, probably in the service of self-esteem protection (23–25). Due to these divergent findings, the use of self-reports is, from a psychometric perspective, especially in young children considered problematic and of limited use. Aspects such as poor reading skills or semantic comprehension can be at least partly resolved by providing reading assistance. There are no available validated self-report questionnaires assessing ADHD symptoms in children under the age of 11 years. Therefore, the present study aims to evaluate the diagnostic accuracy of self-reports in a younger sample.

Objective testing procedures, such as Continuous Performance Tests (CPT), are often part of ADHD assessment. A CPT may comprise a variety of test types. Frequently, sequences of letters, pictures or other stimuli are presented to children, who are then asked to react in certain combinations or to specific stimuli [e.g. CPT, (2)]. It is also notable that there are CPTs that incorporate the Go/No-Go-paradigm [e.g. QIKtest, (1)]. This includes the presentation of stimuli to which the children should react (‘Go’) or inhibit their reaction (‘No-Go’). In addition to reaction times, the types of error (omission and commission) and the extent of selective attention are also recorded in both tasks of objective tests. Discrepancies emerge in assessment technology: while most of the tests are PC-based [e.g. CPT, (2)], others utilize alternative hardware, such as Gameboys [e.g. QIKtest, (1)], with the objective of enhancing compliance through augmented mobility during the test.

CPTs are considered to enhance the accuracy of diagnostic outcomes, diminish diagnostic errors, and furnish data on ADHD symptom severity with lower risk of bias and higher accuracy (26, 27). However, it was shown that these tests lack the capacity to differentiate between disparate etiologies, such as inattention due to anxiety (28). Another limitation of the current diagnostic procedures is that not all cardinal symptoms of ADHD are captured. While inattention is thought to be reflected by delayed reaction times and omission errors, and impulsivity by commission errors, CPTs often fail to recognize the motor restlessness that is also a hallmark of the disorder (29). In consideration of the aforementioned factors and the inadequate validity, CPTs are recommended not be utilized as standalone diagnostic instruments (27).

Associations between the above-mentioned types of methods have previously been examined by several correlational studies (7, 8, 21, 30). Their results ranged from small associations between teacher and parent ratings to strong associations between parental ratings of cardinal ADHD symptoms with omission errors in CPTs (7). Given these methodological differences and the ongoing debate about the best CPT implementation, this study aims to compare the usefulness of the Go/No-Go-paradigm and alternative hardware for diagnostic classification in order to clarify whether these approaches offer added value over established CPT formats.

The recording of these multi-method parameters in the diagnostic process ultimately requires a decision on the weighting of the results during interpretation. The current state of research does not yet provide any clear conclusions on this issue. There are approaches, such as that of Martel et al. (30), which suggest that a symptom should be considered present if either endorsed by the parent or the teacher (‘or’ algorithm), or by both (‘and’ algorithm), or by an ‘averaging’ algorithm. This might reduce systematic measurement error of individual raters (30). However, it is imperative to acknowledge that the augmentation of information sources inherent in multi-method approaches concomitantly necessitates a greater temporal investment and the utilization of personnel with a high level of qualification. This, in turn, can engender protracted waiting periods during which children remain undiagnosed and consequently receive a more circumscribed range of care services. This, in turn, requires healthcare providers to prioritize the most accurate screening tools available.

It is important to distinguish between multimethod diagnostics (MTMM) and related procedures such as performance validity testing. This study focuses on MTMM measures based on several different sources of information in order to substantiate findings and create a more comprehensive clinical picture. The above-described different measures are compared with respect to their indices of diagnostic accuracy to predict a clinical judgment based on the ICD-10 criteria. Furthermore, the extent to which diagnosis can be enhanced by gradually integrating multiple indices in the sense of the ‘and’ algorithm (30) will be investigated. To the best of our knowledge, there are no cross-validation studies that have examined and compared the diagnostic accuracy of multi-perspective and multi-method ADHD diagnoses in children in this way.

## 2 Methods

### 2.1 Participants

*A priori*, a sample size calculation was conducted for a two-sided test with *α* = .05, *β* = .80, and the highest literature-based effect-size measure for multimethod agreement, Spearman’s rho of *ρ* = 0.246 (cf. 21), using G*Power 3.1 (31) to determine a required sample size of *N* = 131. In order to account an estimated rate of 20% dropouts based on previous research (32, 33), 160 children were recruited.

Inclusion criteria were (1) age between 6 and 13 years, and (2) either clear presence or absence of a specialist ADHD diagnosis. This was categorized in the schools on the basis of the information provided by the parents (‘Does your child have an ADHD diagnosis?’). In the context of psychotherapeutic institutions, participation in the study was contingent upon the existence of a pediatric ICD-10 diagnosis of ADHD. The parents were asked a series of socio-demographic questions regarding the child’s age, gender, and the presence of any neurological disorders, partial performance disorders, chronic somatic illnesses, and the child’s regular use of medication. Primary exclusion criteria were incomplete questionnaire sets (missing either parent or teacher questionnaires) or missing consent for teacher involvement, as these reports were fundamental for out multi-methodal diagnostic assessment. Furthermore, to strengthen the psychometric validity of the assessment tools, we planned to exclude cases with severe neurological or global developmental disorders (e.g., IQ <70) or autism spectrum disorder (ASD), as their qualitatively distinct core pathology can confound the measurement of ADHD-specific symptoms.

Our surveys in demographic and clinical settings yielded data from 158 children. Hundred children were recruited at public schools, 58 children were included at the in- and outpatient settings based on a pediatrics given ADHD diagnosis. The study was advertised in schools and in-/outpatient settings. Parents who expressed interest in participating contacted the researchers and were eligible to enroll in the study with their children, provided they met the established inclusion criteria. In the course of the school survey, which was conducted on a class-by-class basis, children who did not participate in the survey were offered the opportunity to engage in alternative activities during the project morning.

Four data sets were excluded due to aborted CPT (n = 3) and QIKtest (n = 1). Further thirteen children from the school survey and one child of the inpatient survey were excluded from the study due to missing parent report questionnaires, which meant that no information was available on a possible diagnosis of ADHD or child’s medication. In the outpatient survey one case needed to be excluded because of a missing teacher report questionnaire. However, no cases were excluded based on the planned exclusion criteria of ASD or severe neurological or global developmental disorders in the final sample. A total of three children diagnosed with ADHD participated in the school surveys and did not take stimulant medication, so it was ensured that the objective measurements remained uninfluenced and could be incorporated into the analysis.

Complete datasets could be obtained from 125 children, including 56 with ADHD (*M_age_* = 9.7 years, *SD* = 2.09, 42 boys [75%]) and 69 children without ADHD (*M_age_* = 9.04 years, *SD* = 2.05, 31 boys [45%]). Our school surveys included three children with dyslexia, one child with Tourette’s syndrome and one child with asthma. The distribution of ADHD diagnoses in our sample is as follows: 26 children received F90.0 (20.8%), 24 children received F90.1 (19.2%), and six children received F98.8 (4.8%). 38 (67.9%) of the children with ADHD were on stimulant medication. None of the children had been diagnosed with a neurological disorder. Information regarding psychiatric medication, comorbidities, partial performance disorders and chronical disorders is reported in **Table 1**. Further details regarding the psychiatric medication and comorbidities can be found in the **Supplementary Material** (**Supplementary Tables S1**, **S2**). Based on the final sample size of N = 125, the actual statistical power achieved for detecting the originally assumed correlation of ρ = 0.246 was 1−β = 0.795 according to a *post-hoc* power analysis, again using G*Power 3.1 (31).

**Table 1:** Demographic and ADHD sample characteristics.

Characteristics	Demographic	ADHD
Psychiatric medication	0	38 (67.9%)
Comorbidities	1 (1.4%)	33 (58.9%)
Partial performance disorder	3 (4.3%)	12 (21.4%)
Chronical disorders (e.g. asthma)	1 (1.4%)	6 (9.3%)

### 2.2 Materials

**Figure 1:**
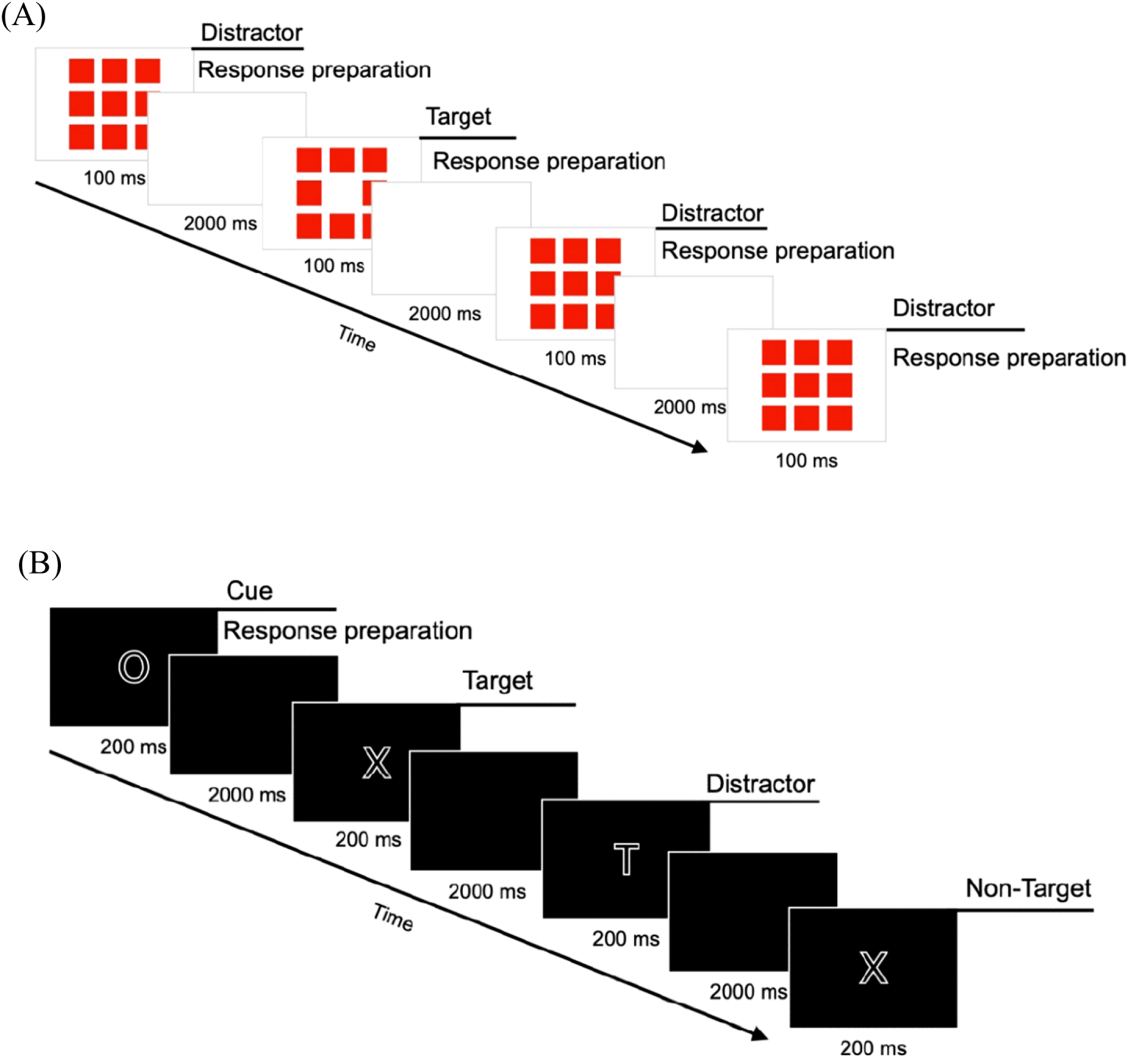
Trial schemes of QIKtest **(A)** and CPT **(B)**.

Seven indices were chosen to quantify ADHD symptoms in the sample, derived from three international standard questionnaires translated to German (34) and two CPTs. The attention problems (AP) subscales from the Child Behavior Checklist 6-18R (CBCL) served as parent report, the Teacher’s Report Form 6-18R (TRF) as teacher report, and the Youth Self Report 11-18R (YSR) as children’s self-report. The wording of the items has not been changed, but taken from the originals. As a possible confound, the three AP subscales differ in the number of items as well as in contents. In contrast to the CBCL and the YSR, the AP subscale in the TRF is divided into two areas: inattention and hyperactivity/impulsivity. In order to circumvent methodological artefacts, an equal number of items and, above all, items with equivalent content were selected for the analysis from each questionnaire. As illustrated in the **Supplementary Material** (see **Supplementary Table S3**), a total of 7 items from each, the CBCL, TRF and YSR AP scale was utilized to assess inattention (IA). This selection was made in order to avoid methodological bias in three ways. Differences in test length, confounds with psychological features other than attention and sample dependence of norms may detract from a fair comparison. Planned *post-hoc* analyses were conducted to examine the effects of these modifications, as the scales were recorded in their original form. At the CPT evaluation level, we included commission errors (action errors, CE) as well as omission errors (skipping errors, OE) in the analysis, as both were supported by previous evidence. Elevated CE have in earlier research been shown to be related to impulsivity, whereas OE were related to inattention (35, 36). In our study, we utilized the PC-based CPT version of Knye et al. (2) and the QIKtest (1), which emulates the Test of Variables of Attention (T.O.V.A.; 37). The trial schema of both objective measurements is illustrated in **Figure 1**.

#### 2.2.1 Assessment methods

##### 2.2.1.1 Parent report questionnaire

IA were assessed using the CBCL AP scale as parent report questionnaire (34). To this end, the original 10 items from the AP scale were used. Parents, were requested to assign a rating to each item on a 3-point-scale: 0 (= not applicable), 1 (= sometimes applicable) and 2 (= exactly or frequently applicable). Answering the items took about 5 minutes. The items were then added together to form a total score of IA. Higher scores indicate greater IA.

##### 2.2.1.2 Teacher report questionnaire

Teachers were asked to rate the items of the AP subscale of the TRF (34). For assessing IA, the original 26 items of the AP subscale were used. The 3-point-scale response levels and interpretations were the same as those used in the CBCL. It took again about 5 to 10 minutes to complete.

##### 2.2.1.3 Self-report questionnaire

Children themselves were invited to complete the subscale AP of the YSR (34). Children were asked to complete the nine items of the AP subscale. The 3-point-scale response categories and interpretations were the same as those used in the CBCL and TRF. Processing took approximately 5 minutes. The YSR, is validated and standardized from the age of 11. As there are no available validated self-report questionnaires for children below 11 years, and as recent research has shown that children at the age of six years can assess their behavior better than previously assumed (21), we used the YSR as a self-report measure for the entire sample (6–13 yrs).

##### 2.2.1.4 Continuous Performance Test

The Continuous Performance Test (CPT; 2) is a well-established and validated computer-based measure of attention (selective and sustained) and impulsivity in children using a n-back task. Five different stimuli (H, O, T, X, Z) are presented on a computer screen in random order [cf. (**Figure 1B**)], and participants are asked to respond to a specific combination of stimuli (O-X) by pressing a key (space bar) with a hand of their choice while ignoring all other letter sequences (distractors). Standardized instructions were made across participants by the CPT system, and in cases where children were experiencing difficulties with reading, the instructions were read aloud by the test instructor. After 200 of the 399 stimulus presentations there is a pause, which can last as long as desired, but in this study, it was limited to a maximum duration of 2 minutes. The test takes approximately 15 minutes.

##### 2.2.1.5 QIKtest

The ‘QIK CPT Visual’ of the QIKtest (1) was included. This gameboy-administered test was developed as an evaluation tool for ADHD interventions, e.g. neurofeedback, for assessment of attention (sustained and selective) and impulsivity. The psychometric criteria of this test had not yet been investigated earlier. It includes visual stimuli in a Go/No-Go task, which are presented on the gameboy-like measuring instrument, thus enabling mobile and computer-independent implementation and allowing more flexibility in implementation. Either 8 or 9 red boxes light up in the center of the device. The children have to press a button if 8 boxes are flashing; if 9 boxes are lit up, they should not press a button [cf. (**Figure 1A**)]. Accordingly, the instructions were standardized and administered prior to the commencement of the test. The instructions were as follows: ‘Press one of the two black buttons when eight red boxes flash. If nine boxes flash, do not press any button.’ Even if there is a differentiated consideration of up to four test parts during the evaluation, the test is carried out without breaks. The duration of this test is 21 minutes.

### 2.3 Procedure

The Ethics Committee of Bielefeld University approved this research (EUB-2023-046-S). Informed consent was ensured through personal explanations of the study content and written consent from participating parents, teachers and children. This study’s design, hypotheses and analytical plan were preregistered after data collection had started, but before analyses were undertaken (see #194513 https://aspredicted.org/b7vy-6pt4.pdf).

#### 2.3.1 General procedure

Data collection was conducted from April 2023 to November 2024 at five public schools, the pediatric wards of LWL Hospital in Marsberg and Paderborn, and the Neuropsychological Outpatient Clinic at Bielefeld University. We recruited children with a pre-existing ADHD diagnosis made by a child and adolescent psychiatrist and control children without mental disorders. The diagnosis was therefore always made independently and prior to actual participation in the study. Parents or guardians provided written informed consent for themselves and their children. At all settings, data collection was carried out in small groups of up to eight children, supervised by at least one or more test instructors. The procedure was explained to the children and their verbal consent was obtained, with confidentiality assured for all partici-pants through the use of codes. The CBCL and socio-demographic questions were completed by the parents separately from the project survey, either in paper form in 'parent document' folders or digitally via Qualtrics LLC (QR code or URL link). After completion of data collection, a discussion about workplace design and concentration exercises was conducted in all settings.

#### 2.3.2 Procedural differences according to setting heterogeneity

Due to the different clinical and school conditions, specific details of data collection had to vary, which was deliberately accepted in order to ensure broader ecological validity. With regard to the test sequence, randomization of CPT, QIKtest and YSR was carried out in the school setting. In outpatient and inpatient settings, however, only CPT and QIKtest were randomized. This randomization was additionally restricted in cases where a break was required for medication intake.

With regard to medication control, the central requirement in all settings with ADHD children was to ensure that CPT and QIKtest were carried out without medication. In the school setting, this was ensured via the parent report. In the clinical settings, the staff of the facility took over this control, whereby medication was taken, if indicated, during a break immediately after the CPT and QIKtest had been carried out.

The return of the TRF also differed: while the TRF in the school setting was completed and returned by the teacher immediately after the data collection session, in the clinical settings it was returned by post by the clinic or residential care staff and usually arrived after the project survey of the remaining parameters had been completed. The diagnostic status (ADHD vs. non-ADHD) of the children in the school setting was also only determined after the parental questionnaires had been returned.

### 2.4 Data analysis

Data were analyzed using IBM SPSS Statistics (Version 28.0.1.0, 38). The questionnaire and test data were compiled and entered into a database in the SPSS statistics program. Missing data in the questionnaires were addressed using multiple imputations based on a fully conditional specification MCMC algorithm [e.g. (39)]. There was a total of 13 missing items across TRF (n = 3) and CBCL (n = 10). Despite this small number of missing responses, a conservative imputation strategy was pursued to increase statistical robustness. Therefore, we generated 15 imputed datasets with a maximum of 50 iterations.

For the IA indices of CBCL, TRF, and YSR, the seven items were each aggregated to create a composite index score. The CE and OE of QIKtest and CPT were also added together. By adapting the questionnaires to a reduced number of items from the original attention problems subscale, internal consistencies (Cronbach’s alpha) were calculated to test the reliability.

#### 2.4.1 Indices of ADHD classification accuracy

Our research question is addressed through Receiver Operating Characteristic (ROC) analysis, providing a variety of information for calculating indices for test’s classification performance beyond sensitivity and specificity. Both sensitivity and specificity can be interpreted using the standardized qualitative evaluation approach developed by Glascoe (40). Prior studies on classification accuracies of ADHD measurements [e.g. (41)], have predominantly employed the Area under the Curve (AUC) to ascertain the most accurate measurement instrument including information, incorporating data from the entire ROC-curve. The interpretation of AUC values can be facilitated by utilizing the framework provided by Hosmer et al. (42). In contemporary research on diagnostic classification performance, the Diagnostic Odds Ratio (DOR) is being utilized with increasing frequency [e.g. (43, 44)]. A key benefit of the DOR is its independence from the prevalence of the targeted disorder, thereby enabling enhanced comparability in meta-analyses and analogous studies. The DOR is interpreted as follows: A DOR of e.g. 2 means that the odds of having a particular disease are twice as high with a positive test result as with a negative test result.

Consequently, we conducted ROC analyses including AUC for the IA scores of CBCL, TRF and YSR, as well as the CE and OE of CPT and QIKtest, incorporating the methodology for calculating the DOR as outlined by Glas et al. (45; formula in **Supplementary Equation 1**). To enhance the validity of our results, the analyses will also be carried out using the original AP scales. In order to explore whether the diagnostic accuracy of two indices differs from each other, *post-hoc* pairwise comparisons of all AUCs of the seven indices were performed according to DeLong et al. (46). To ensure the integrity of the results, multiplicity control is a prerequisite. Due to the high number of comparisons in the pairwise AUC comparisons, the false discovery rate (FDR) according to Benjamini-Hochberg is used. A further analysis examined the covariates: setting, gender, age and comorbidities. A covariate-adjusted ROC analysis (COAROC; 47) was performed using binary logistic regression to estimate the predicted probabilities of the covariates and the index variables. Due to the large number of variables included, bootstrapping (m = 1000) was employed. The ‘bias-corrected and accelerated’ method was used to calculate the 95% confidence intervals (CI). In cases of disagreement regarding statistical significance between the p-value and the CI, the CI was chosen as the more robust method. In this sub analysis, significance applies if the CI does not contain the value 0.

#### 2.4.2 Application of the ‘and’ algorithm

According to the ‘and’ algorithm by Martel et al. (30), the seven indices recorded (IA by CBCL, TRF, and YSR, as well as CE and OE by CPT and QIKtest) are combined in a step-by-step process. This is achieved by employing the cut-offs that have been determined in the ROC analysis: CBCL ≥ 4.5, TRF ≥ 4.5, YSR ≥ 5.5, CPT OE ≥ 3.5, QIKtest OE ≥ 27, CPT CE ≥ 7.5, QIKtest CE ≥ 34.5. The cut-offs were determined as coordinate points from the ROC analysis based on the calculated sensitivities and specificities. The subsequent formation of individual combination variables is predicated on the results of the diagnostic accuracy analyses, with the index exhibiting the highest accuracy being included first, and the index exhibiting the lowest accuracy being combined last. These are represented by the coding ‘cut-off exceeded’ (=1) vs. ‘not exceeded’ (=0). Chi-square tests are calculated for each of these seven stepwise composite variables. In each stage of the process, the cut-off variable of the indices is compared with the criterion of the specialist diagnosis ‘ADHD’ (=1) versus ‘not ADHD’ (=0).

In addition, a *post-hoc* analysis will examine the extent to which the modified scale composition with a focus on IA deviates from the original AP scale. To this end, the approach already described here will also be calculated using the full questionnaire scales and then compared with the IA scales’ index combination.

## 3 Results

Prior to the analysis of the main research questions, the internal consistencies of the three questionnaire indices (CBCL-IA, TRF-IA, and YSR-IA) were determined using Cronbach’s α. The analysis revealed internal consistencies (Cronbach’s- α) ranging from 0.827 (CBCL-IA) and 0.826 (TRF-IA) to 0.737 (YSR-IA). For the subgroups examined in the YSR-IA, YSR_11-13_ α = .744 and YSR_6-10_ α = .730 were obtained. Accordingly, sufficient reliability can be assumed for further analyses. It was determined that none of these indices contained items whose omission would have resulted in an increase in internal consistency. Applying the interpretation of Streiner (48), very good (CBCL-IA, TRF-IA) and good (YSR-IA) internal consistencies were found. In comparison with the reliabilities from the standardization (34) of the AP scale (CBCL_field sample_ α = 0.74, TRF_field sample_ α = 0.93, YSR_field sample_ α = 0.70-0.74), the excerpts employed in our study with a focus on inattention demonstrate only minimal variation.

### 3.1 Index accuracies

**Figure 2:**
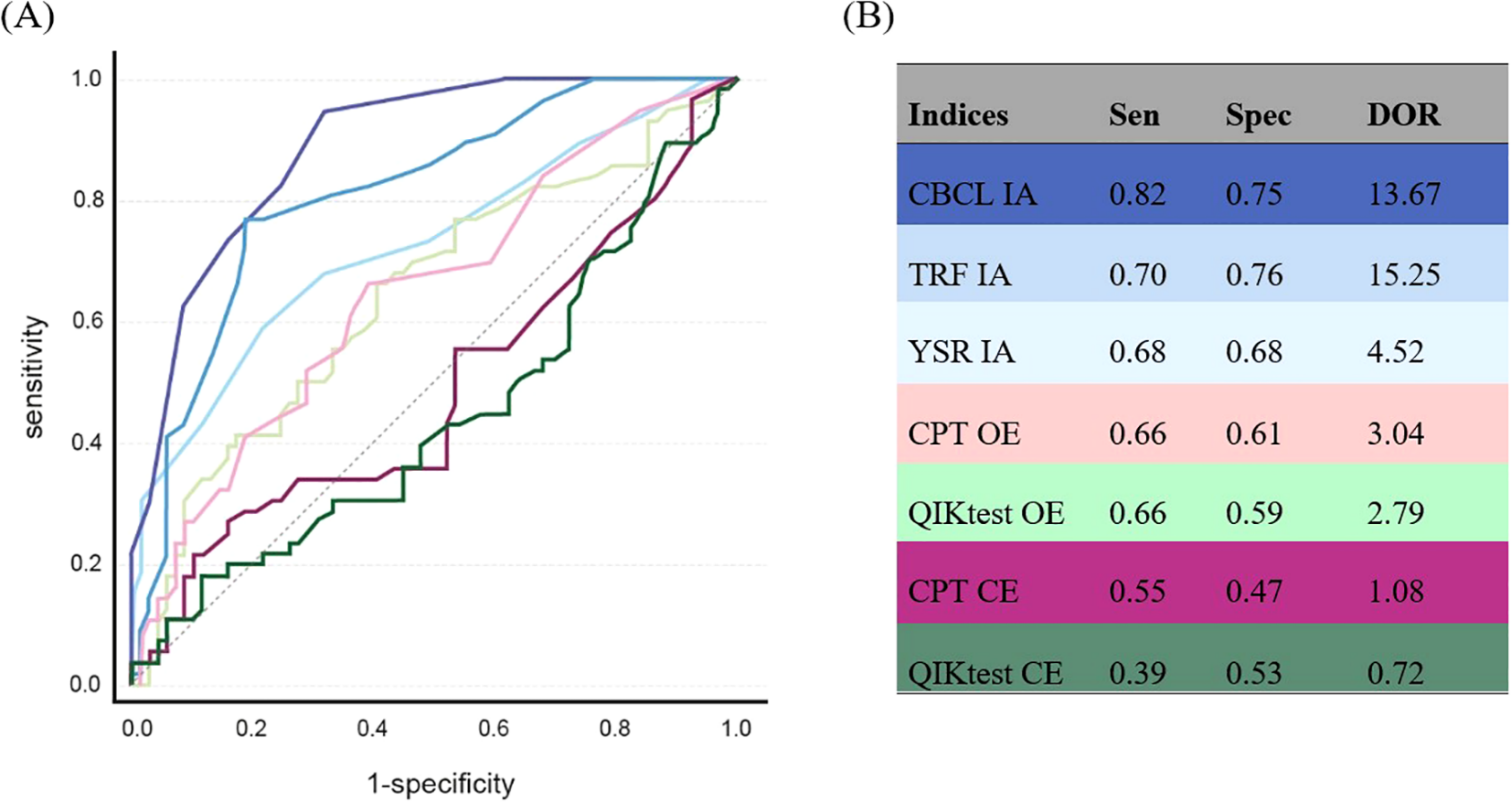
ROC analysis of the ADHD symptom measurement instruments. **(A)** ROC curves: dark blue = CBCL IA, medium blue = TRF IA, light blue = YSR IA, light pink = CPT OE, dark pink = CPT CE, light green = QIKtest OE, dark green = QIKtest CE, grey line = reference line. **(B)** index values of ROC analysis: Sen = sensitivity, Spec = specificity, DOR = Diagnostic Odds Ratio, IA = inattention, OE = omission errors, CE = comission errors. Sen & Spec belong to the depicted index-values. The ROC analysis with the original scales yielded the following results: CBCL-AP: AUC:.920 Sen:.82 Spec:.88 DOR: 33.41, TRF-AP: AUC:.791 Sen:.77 Spec:.68 DOR: 7.11, YSR-AP: AUC:.726 Sen:.68 Spec:.68 DOR: 4.52.

As depicted in **Figure 2A**, the results of the ROC analysis revealed an AUC of.889 for CBCL-IA (95% *CI*: 0.834-0.943, *p* <.001), an AUC of.817 for TRF-IA (95% *CI*: 0.742-.891, *p* <.001), and an AUC of.726 for YSR-IA (95% *CI*: 0.635-0.817, *p* <.001). Furthermore, an AUC of.647 for OE in CPT (95% *CI*: 0.549-0.744, *p* = .003), an AUC of.643 for OE of QIKtest (95% *CI*: 0.544-0.741, *p* = .005), an AUC of.486 for CE of CPT (95% *CI*: 0.381-0.590, *p* = .788), and an AUC of.441 for CE of QIKtest (95% *CI*: 0.338-0.544, *p* = .260). In addition, CBCL-IA, TRF-IA and YSR-IA showed statistical superiority over a random classifier with regard to their capacity for classification. The OE of the CPT also achieved marginal significance. As shown in **Figure 2B**, the CBCL-IA showed optimal values for AUC, DOR, and sensitivity compared to the other instruments. In terms of specificity, the TRF-IA achieved the highest score. The TRF-IA showed slightly superior performance in terms of AUC and sensitivity. A modest disparity was observed between the sensitivities and specificities of the YSR-IA and the OE of the CPT and QIKtest. This pattern also emerged in the calculations for the original AP scales of the three questionnaires. The comparison between the AP and IA scales revealed a marked difference, particularly in the CBCL’s specificity and DOR. In the TRF, the indices showed higher diagnostic classification performance in IA than in AP. Nevertheless, the pattern of results remains the same: the CBCL and TRF achieved the highest scores in the index values, followed by the YSR.

A more pronounced discrepancy emerged when assessing the AUC. While the OEs of the two objective tests were comparable in terms of specificity, with the QIK exhibiting a marginal inferiority, a more pronounced discrepancy emerged between the OE of CPT and QIKtest and the CE of the two tests. Additionally, the two test procedures diverged in this error category, with the CPT attaining more favorable values in AUC, sensitivity, specificity and DOR. Nevertheless, it is important to note that the CE of both tests produced ROC curves nearly equal to random observations. In order to consider the fact that, three children in the school setting were labeled with ADHD without being confirmed by a pediatric for the participation in our study, the ROC analyses and index calculations were repeated on a comparative basis, excluding these three cases. This resulted in only minor changes with the same pattern of results, so that further calculations were carried out with the total sample (**Supplementary Table S6**).

The *post-hoc* pairwise AUC tests showed a similar pattern like our main result. The CBCL-IA differed significantly from all the other indices (*p* = .002 to *p* = .01). Comparisons of the TRF-IA revealed significant differences with all indices except the YSR-IA. However, the YSR-IA showed exceptionally no significant difference from CPT OE (*p* = .26) and QIKtest OE (*p* = .27). The other pairwise comparisons can be found in **Supplementary Table S4**. The results of the COAROC are presented in **Supplementary Table S6**. Examination of the 95% confidence intervals revealed that ‘setting’ was a statistically significant predictor (*B* = 91.01, 95% *CI* [36.60; 112.17]; *p* <.001). However, all other predictors, including CBCL-IA, TRF-IA, YSR-IA, OE and CE from the CPT and QIKtest, as well as the other sociodemographic variables, showed no significant influence as their 95% confidence intervals included zero.

### 3.2 Age comparison of self-report

As the YSR-IA was in our study also completed by children under the age of 11, we additionally analyzed how accurately these group of children was able to assess their symptoms using the YSR-IA. ROC analyses were separately conducted for two age groups (6–10 and 11–13 years), and the same indices were obtained as for the main question. Comparison of the age groups (**Table 2**) revealed that the older children (between 11 and 13 years) achieved higher values in AUC, sensitivity, specificity and DOR. The ROC analysis of the data from younger children resulted in lower values than in the cross-age analysis. Nevertheless, significant classification performance was found for both age groups, with greater accuracy for the self-reports of the children in the older age group.

**Table 2:** Index values of the age groups from the YSR-IA.

Indices	n	Sen	Spec	AUC (95% CI)	DOR
YSR IA	125	0.68	0.68	.726** (0.635-0.817)	4.52
YSR 11-13	41	0.94	0.74	.820** (0.689-0.952)	5.51
YSR 6-10	84	0.89	0.83	.683* (0.566-0.800)	1.66

Note: YSR IA represents the results for the YSR items on inattention in total, whereas YSR 11–13 and YSR 6–10 depict the age group respectively. *p <.01, **p <.001.

### 3.3 ‘And’ algorithm

The incremental incorporation of our seven indices yielded a diagnostic benefit (**Table 3**). Initially, the CBCL-IA correctly and significantly (p <.001) identified 47 out of 56 ADHD cases. However, the subsequent incorporation of TRF-IA and YSR-IA resulted in a further enhancement in sensitivity. The number of correctly identified cases of ADHD rose to 55, thereby reducing the number of false negative cases to a single instance. Conversely, the subsequent integration of computer-based performance tests (CPT and QIKtest) demonstrated no additional enhancement in sensitivity for either error index (OE and CE); the number of correctly identified ADHD cases remained constant. Conversely, the incorporation of these tests resulted in a substantial decline in specificity, as evidenced by a persistent escalation in false-positive outcomes. The number of false-positive cases increased from 17 with the CBCL-IA to 58 with the final combination with the QIKtest CE.

**Figure 3:**
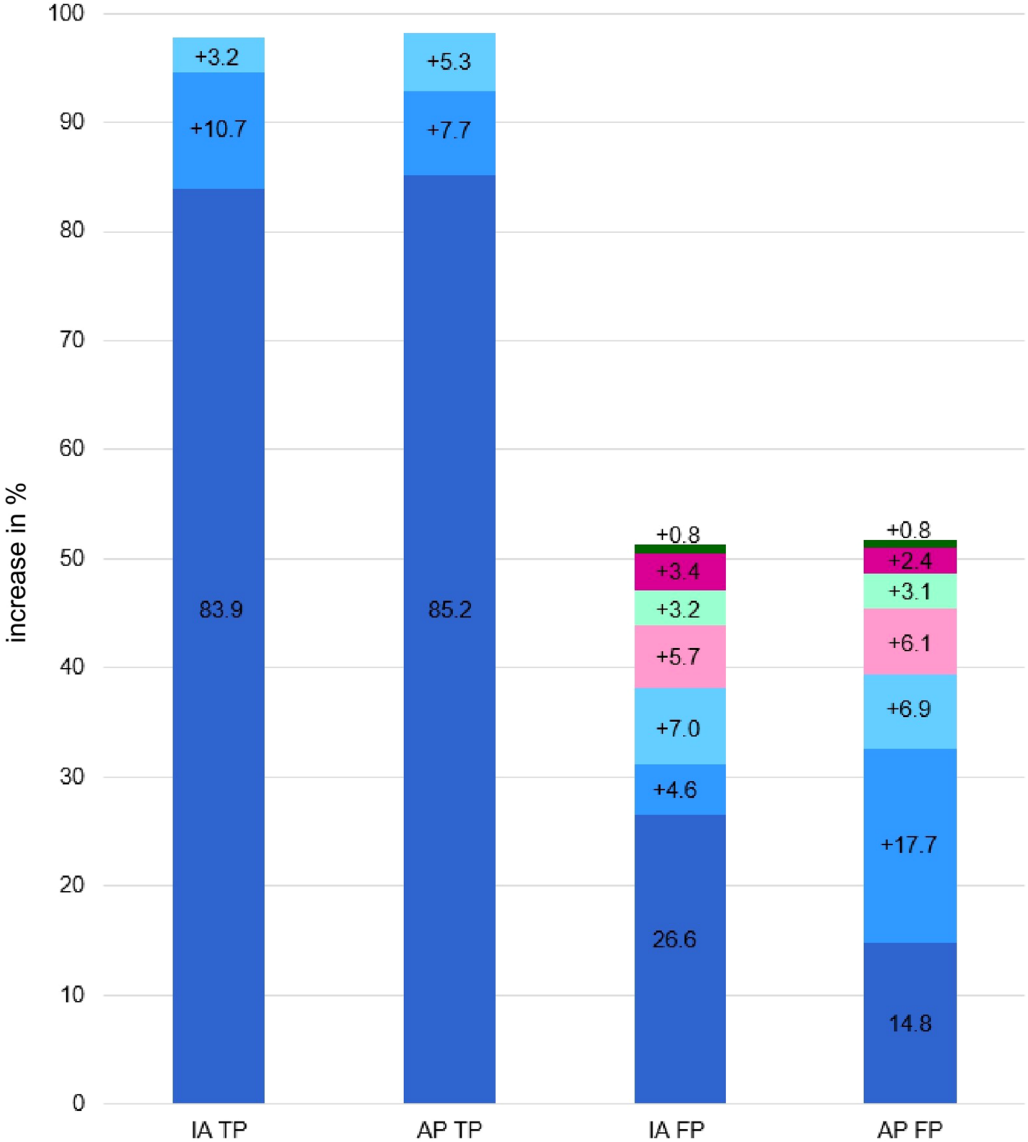
Comparison of index-combination with True Positives and False Positives of IA and AP scales. The figures show the percentage shares that are added when the indices are combined. If there is no increase in value, the last method used is displayed. IA = Inattention scale, AP, Attention Problem scale, TP, True Positives; FP, False Positives. Dark blue = CBCL IA; medium blue = TRF IA; light blue = YSR IA; light pink = CPT OE; dark pink = CPT CE; light green = QIKtest OE; dark green = QIKtest CE.

The comparative *post-hoc* analysis between the application of the ‘and’ algorithm in IA and AP revealed a similar pattern (see **Figure 3**). The stepwise classification of TPs was almost identical. For FPs, there was a larger proportion at the beginning due to the IA of the CBCL, after which the remaining indices in combination contributed smaller proportions to FP percentages. For FPs of AP, the proportions were more evenly distributed, but tended to decrease at higher combination levels. IA and AP ended up with the same final results for TP and AP.

**Table 3:** Stepwise multi-method index-combination.

Index-combination	ADHD (n = 56)		Non-ADHD (n = 69)	
	TP	FN	TN	FP
CBCL-IA**	47	9	52	17
+TRF-IA**	53	3	45	24
+YSR-IA**	55	1	35	34
+CPT OE**	55	1	26	43
+QIKtest OE**	55	1	20	49
+CPT CE*	55	1	13	56
+QIKtest CE*	55	1	11	58

Note. TP, True Positive; FN, False Negative; TN, True Negative; FP, False Positive; N, 125, *p <.01, **p <.001.

## 4 Discussion

In our study, we aimed to determine the most accurate diagnostic instrument for ADHD in children by examining various assessment methods. As a secondary objective, to challenge the multimethod approach, the ‘and’ algorithm was utilized with the seven indices that have been the focal point of this study.

### 4.1 Exploration of the shortened version of AP scale

The decision to utilize the IA subscale comprising seven equivalent items instead of the full AP scale was a critical methodological choice. But it was necessary for achieving direct comparative analysis across the CBCL, TRF, and YSR using content-identical statements in different report formats (parent, teacher, self-report). This approach ensures enhanced methodological comparability when examining the instruments’ contribution to the diagnostic algorithm.

To assess the impact of this modification, we systematically compared the shortened IA scales with the original full AP scales. The internal consistency estimates showed only minor deviations. In the ROC analysis, the TRF-IA demonstrated a higher DOR compared to the TRF-AP, suggesting a potential gain in overall diagnostic accuracy for the teacher report. Conversely, the CBCL-IA showed slightly lower specificity and DOR than the CBCL-AP. Further research should therefore examine whether the focused application of identical, abbreviated IA scales across different reporting formats can increase not only methodological comparability but also clinical efficiency and specificity in the diagnostic process.

Crucially, in the analysis of the stepwise diagnostic approach (the ‘and’ algorithm), no substantial difference emerged when using either the IA or the AP scales. This finding confirms the robustness of our main conclusion, demonstrating that the choice of the shortened IA scales did not compromise the validity of the combination model. This comparative *post-hoc* analysis ultimately justifies the use of the streamlined IA scales for achieving coherent cross-instrument comparisons within a unified diagnostic framework.

### 4.2 Assessing ADHD with questionnaires and objective tests

CBCL-IA and TRF-IA were clearly superior to the other indices in ROC analysis based on various parameters. CBCL-IA due to high values for AUC and sensitivity, TRF-IA due to high specificity and DOR. *Post-hoc* analyses also showed that CBCL-IA differs significantly from all other indices, with TRF-IA only showing no significant difference from YSR-IA. These results align strongly with conclusions in recent literature, which also highlights the CBCL for sensitivity and TRF for specificity (4, 49). The lower diagnostic accuracy of the YSR compared to CBCL and TRF is consistent with familiar findings, which hypothesize that the finding may be due to the young age of participants or the nature of ADHD symptoms impairing their ability to accurately report externalizing behavior (50).

Regarding QIKtest and CPT, we observed lower diagnostic accuracy for measures of OE and CE compared to CBCL-IA, TRF-IA and YSR-IA. This suggests that these objective measures may not be sufficiently specific to ADHD symptoms. Previous research has reached conflicting conclusions on this. While some studies indicate that objective tests are more robust, less biased, with more replicable results [e.g. (26)], and can effectively distinguish between ADHD and non-ADHD (51), other studies with similar findings to ours (52) question the validity. However, when compared to the clinical diagnoses, the CE in the CPTs did not demonstrate greater diagnostic accuracy than chance, supporting the notion, that CPTs should not be employed as the exclusive diagnostic procedure, as is often reported in the literature (4, 52).

To improve the diagnostic accuracy of objective tests, two recent studies investigated alternative indices for the performance of objective tests (28, 53). Instead of OE as a standalone parameter, Hult et al. (53) additionally calculated indices for assessing inattention based on reaction time and reaction time variation (AUC = 0.74). Furthermore, Berger et al. (28) used the average number of correct responses across all test trials to measurement of the children's attentional focus on the target stimuli independent of reaction times, CE, or OE (AUC_7year-olds_ = 0.91, AUC_12year-olds_ = 0.75). Further research should pursue and expand on these promising initial results.

The robustness of our results was supported by the calculation of COAROC analysis. There were no significant influences from age, gender, or comorbidity. This is surprising, as the age comparisons from the YSR-IA showed a significant difference between children aged 6–10 and 11–13 with higher diagnostic accuracies for the older children. Accordingly, the significant effect of age on the YSR-IA, when viewed in isolation, appears to be explained by other variables when viewed in a multivariate analysis. But the COAROC showed significant influences from the covariate ‘ setting’, representing a limitation of the study.

### 4.3 Assessing ADHD symptoms using the ‘and’ scoring algorithm

In the second part of our study, based on a stepwise ‘and’ algorithm, the analysis showed that the combination of CBCL-IA, TRF-IA and YSR-IA represents the clinically optimal compromise, achieving the maximum possible sensitivity (98.2%). Continuing the algorithm by adding objective test indices (OE and CE) serves as a clear negative example of over-optimization. Although sensitivity cannot be further increased, each subsequent addition leads to a reduction in specificity. This reverses the pattern (fewer TN, more FP) and increases the FP rate to an unacceptable level. The efficiency of the algorithm is therefore only given up to the inclusion of the YSR-IA. This combination is ideal as a sensitive tool. The main task for future research is to improve the moderate specificity of this trio combination. This should be achieved by using weighted algorithms and comparing their results to the binary ‘and’ algorithm.

The main practical takeaway from our findings is the clear diagnostic superiority of information derived from parent and teacher questionnaires (CBCL-IA and TRF-IA) for the most accurate diagnosis of ADHD in children. Conversely, the addition of objective tests like CPTs not only provides little added value but actively harms the diagnostic process by drastically reducing specificity and increasing the rate of False Positives (FP). This suggests a strong recommendation against their routine use as a primary diagnostic tool, reinforcing that they should not be employed as the exclusive diagnostic procedure, as they risk overdiagnosis.

### 4.4 Limitations

Our study design involved several critical limitations that should be considered. First, diagnostic asymmetry presents a limitation because while the clinical diagnosis of ADHD was obtained independently and blinded for all participants, we cannot completely exclude the possibility of undiagnosed ADHD among children included in the school survey. This is due to the fact that the control group status was based on parent reports regarding the non-existence of a hyperkinetic disorder, rather than a specialist diagnosis as was the case for the clinical groups. For future research, a longitudinal design would be preferable, in which a non-selected sample is examined and subsequently monitored to ascertain which individuals have received a diagnosis. Further confounding factors must be considered, including but not limited to for example access to institutions and socio-economic factors.

Second, the setting heterogeneity represents a major methodological challenge. Although the introduction of setting-specific protocols should increase validity, the differences in medication control and test order necessitate careful interpretation. The varying control mechanisms for stimulant intake and differing return processes for TRFs (directly in person after data collection and via post) may have introduced subtle biases into the performance and questionnaire data, respectively. Future studies should therefore ideally aim for a completely uniform protocol to ensure that the effects observed are primarily attributable to the sample and not to procedural differences.

Third, the exploratory use of the YSR-IA for children aged 6 to 10 must be acknowledged as a psychometrically weak choice. Although the YSR-IA has been primarily validated for individuals aged 11 and older, our decision aimed to capture the rarely studies self-report-perspective of younger children. Our analyses of internal consistency support the reliability but not necessarily the full validity in this exploratory age range. This finding underscores the complex nature of self-assessment in children and reinforces the need to develop age-appropriate and validated assessment tools in the future. Future research should further clarify the full validity of the YSR-IA in this exploratory age range.

Fourth, comparative analyses between the AP full scales and our ‘shortened’ IA scales provide additional insights. While the reduction of the AP scale to IA resulted in reduced diagnostic accuracy in the CBCL, there was an increase in the TRF. When interpreting the results, however, this difference should be taken into account, especially with regard to comparisons with the original AP scales used in the literature. Furthermore, this aspect should be investigated in further research: Why does a reduction in the scale items in the parent report lead to a reduction in the diagnostic classification, while in the teacher report it leads to an increase? Especially considering that the items are identical in content.

Fifth, a potential limitation of the present study is that the validity of the objective tests may have been influenced by reduced motivation and compliance, as suggested by qualitative observations in various settings, even though test administrators were able to achieve a low drop-out rate. Furthermore, the inclusion of the QIKtest must be considered a limitation. While its choice was strategic for the comparative nature of this study – to assess differences between Go/No-Go and sequential task format – we acknowledge the missing extensive psychometric validation compared to widely used CPTs. Despite this, CPT and QIKtest achieved similar diagnostic accuracy results, suggesting that differences in task format may not be as pronounced as theoretically expected. This finding, however, must be interpreted with caution due to the QIKtest’s limited validation, which affects the generalizability of our results regarding objective test formats.

Sixth, among recent studies on diagnostic measurements of ADHD in children, for example Martel et al. (30), our study is comparatively small. However, it remains within the upper quartile when compared to extant studies (cf. 7, 8) and relies on the calculation of the *a priori* sample size. The *post-hoc* power analysis also showed that the desired informative value was achieved based on the sample collected.

Finally, we cannot exclude that participant’s awareness of the study’s focus on attention might have subtly influenced their responses, especially in the clinical survey settings. Even though different settings were included, it became apparent that the predominant participation in the settings reflected a pattern of symptom severity (in schools, more children without ADHD; in outpatient clinics, mild to moderate symptom severity; and in inpatient settings, significant to severe symptom severity). Nevertheless, in line with other studies [e.g. (54, 55)], in our study a higher rate of cases was classified as combined and hyperactive-impulsive subtype and a lower rate of cases as predominantly inattentive subtype.

## 5 Conclusion

Our study provides compelling evidence that a diagnostic strategy for ADHD prioritizing parent and teacher reports (CBCL-IA, TRF-IA), along with the inclusion of self-reports like YSR-IA, is superior to a multi-method approach that includes CPT and QIKtest, which risk overdiagnosis by contributing unnecessary FP. Yet, before discarding these tests from routine diagnostics, it should be investigated whether they represent a valuable source of information for other clinical purposes. Further research should focus on optimizing the combined questionnaire approach, perhaps through weighted algorithms, to improve specificity without sacrificing the demonstrated high sensitivity.

## Data Availability

The raw data supporting the conclusions of this article will be made available by the authors, without undue reservation.

## Author’s note

This study has been preregistered: #194513 - A multi-method case-control study of ADHD diagnostics in children (https://aspredicted.org/b7vy-6pt4.pdf).
