# Supplementary material for: Multi-method ADHD diagnostics in children: CBCL and TRF lead the way
Source: Front Psychiatry. 2025 Nov 17;16:1668149. doi: 10.3389/fpsyt.2025.1668149 (PMC12666681; doi:10.3389/fpsyt.2025.1668149)
Supplement: Supplementary file 1 [file Supplementaryfile1.docx]

**Supplementary Material**

**Table S1.** Distribution of the psychiatric medication.

| **Psychiatric medication** | **n** |
| --- | --- |
| Methylphenidate slow-release | 9 |
| Methylphenidate unretarded | 21 |
| Dexamfetamine hemisulfate | 1 |
| Lisdexamfetamine dimesilate | 1 |
| Atomoxetine | 1 |
| Guanfacine | 2 |
| Pipamperone | 2 |
| Risperidone | 1 |
| SSRI | 3 |

**Table S2.** Distribution of comorbid ICD-10 diagnosis.

| **Comorbid ICD-10 diagnosis** | **n** |
| --- | --- |
| F32.1 | 5 |
| F80.1 | 3 |
| F80.2 | 2 |
| F80.8 | 1 |
| F81.1 | 2 |
| F40.1 | 1 |
| F42.2 | 2 |
| F42.1 | 2 |
| F95.1 | 1 |
| F92.8 | 2 |
| F93.1 | 1 |
| F93.8 | 8 |
| F94.1 | 2 |
| F98.1 | 1 |

**Table S3.** Items of Child Behavior Checklist 6-18R (CBCL), Teacher’s Report Form 6-18R (TRF) and Youth Self Report 11-18R (YSR).

| **CBCL** | **TRF^1^** | **YSR** |
| --- | --- | --- |
| 1.Behaves too young for his/her age. | 1.Behaves too young for his/her age. | 1. I act too young for my age. |
| 4. Does not finish tasks he/she has started. | 4. Does not finish tasks he/she has started. | 4. I don’t finish tasks I’ve started. |
| 8. Cannot concentrate, cannot pay attention for long. | 8. Cannot concentrate, cannot pay attention for long. | 8. I have difficulties concentrating or paying attention for longer. |
| 13. Is confused or distracted. | 13. Is confused or distracted. | 13. I am confused or distracted. |
| 17. Daydreams or is lost in thought. | 17. Daydreams or is lost in thought. | 17. During the day I am dreamy or lost in my thoughts. |
| 78. Is inattentive or easily distracted. | 78. Is inattentive or easily distracted. | 41. I do things without thinking. |
| 80. Stares into space. | 80. Stares into space. | 78. I am inattentive or easily distracted. |
| 10. Cannot sit still, is restless, or hyperactive. | 22. Has difficulty following instructions. | 10. I can't sit still for long. |
| 41. Is impulsive or acts without thinking. | 49. Has learning difficulties. | 41. I do things without thinking. |
| 61. Does poorly in school. | 60. Is apathetic and unmotivated. | 61. I'm bad at school. |
|  | 61. Does poorly in school. |  |
|  | 72. Works sloppily. |  |
|  | 92. Does not reach his potential; could do better. |  |
|  | 100. Does not complete assigned tasks. |  |

**Note.** Black = items used in this study to measure inattention gray = additional items from the attention problem scale that were not used in this study. CBCL = Child Behavior Checklist 6-18R, TRF = Teacher’s Report Form 6-18R, YSR = Youth Self Report 11-18R. ^1^ **=** Items from the manual’s inattention section, 12 items associated with hyperactivity are not listed here. German translations (Döpfner et al., 2014) of the listed items were used in our study. The item formulations used here correspond to those in the original questionnaires.

**Equation S1.** Formula for calculating the Diagnostic Odds Ratio (DOR).

DOR = $\frac{TP}{FN}\div\frac{FP}{TN}=\frac{sens}{(1-sens)}\div\frac{(1-spec)}{spec}$

**Note.** TP = True Positives, FN = False Negatives, FP = False Positives, TN = True Negatives, sens = sensitivity, spec = specificity.

**Table S4.** Pairwise AUC tests of the used seven indices (DeLong, 1988).

| **Index pairs** | ***z*** | ***p*** |
| --- | --- | --- |
| CBCL-IA, TRF-IA | -2.91 | <.007* ^FDR^ |
| CBCL-IA, YSR-IA | -3.21 | <.002*^FDR^ |
| CBCL-IA, CPT OE | -4.76 | .02*^FDR^ |
| CBCL-IA, QIKtest OE | -4.70 | .01*^FDR^ |
| CBCL-IA, CPT CE | -7.15 | .006* ^FDR^ |
| CBCL-IA, QIKtest CE | -7.69 | .005* ^FDR^ |
| TRF-IA, YSR-IA | -0.69 | .488 |
| TRF-IA, CPT OE | -2.21 | .039* ^FDR^ |
| TRF-IA, CPT CE | -4.53 | .004* ^FDR^ |
| TRF-IA, QIKtest OE | -2.15 | .043* ^FDR^ |
| TRF-IA, QIKtest CE | -5.05 | .003* ^FDR^ |
| YSR-IA, CPT OE | -1.13 | .26 |
| YSR-IA, CPT CE | -3.27 | <.003* ^FDR^ |
| YSR-IA, QIKtest OE | 1.24 | .27 ^FDR^ |
| YSR-IA, QIKtest CE | -3.99 | <.003* ^FDR^ |
| CPT OE, CPT CE | 2.67 | .042* ^FDR^ |
| CPT OE, QIKtest OE | 0.08 | .938 |
| CPT OE, QIKtest CE | 3.09 | <.004* ^FDR^ |
| CPT CE, QIKtest CE | 0.85 | .397 |
| QIKtest OE, QIKtest CE | -3.37 | .002* ^FDR^ |

**Note.** IA = inattention, CBCL = Child Behavior Checklist 6-18R, TRF = Teacher’s Report Form 6-18R, YSR = Youth Self Report 11-18R, OE = omission errors, CE = commission errors. *p < .01, **p < .001, ^FDR^ = False Discovery Rate for p-adjustments. According to FDR, once the α-level was exceeded, the remaining four comparisons were no longer adjusted.

**Table S5.** Bootstrapped covariate-adjusted ROC-analysis.

| **Variables** | **Coefficient (B)** | ***SE*** | ***p*** | ***CI* [Bca 95%]** | |
| --- | --- | --- | --- | --- | --- |
|  |  |  |  | Lower | Upper |
| CBCL-IA | 6.16 | 2.07 | .007 | -1.21 | 6.86 |
| TRF-IA | 5.23 | 2.26 | .003 | -1.68 | 7.39 |
| YSR-IA | 3.37 | 2.49 | .309 | -3.26 | 5.77 |
| CPT OE | -4.05 | 1.65 | .181 | -5.57 | 0.44 |
| CPT CE | 0.23 | 0.22 | .047 | -0.42 | 0.54 |
| QIKtest OE | 0.09 | 0.17 | .267 | -0.42 | 0.25 |
| QIKtest CE | -0.16 | 0.16 | .257 | -0.43 | 0.21 |
| Age | -1.47 | 3.29 | .686 | -6.89 | 5.47 |
| Gender | 4.08 | 16.28 | .613 | -28.67 | 39.68 |
| Comorbidities | 55.48 | 36.39 | .034 | -48.16 | 112.44 |
| Setting | 91.01 | 19.50 | .001* | 39.55* | 115.96* |
| **Note.** IA = inattention, CBCL = Child Behavior Checklist 6-18R, TRF = Teacher’s Report Form 6-18R, YSR = Youth Self Report 11-18R, OE = omission errors, CE = commission errors, SE = standard error, CI = Confidence Interval. Bootstrapping with m = 1000.  * = significance applies if the CI does not include 0. | | | | | |

**Table S6.** Comparison of ROC analysis with and without the ADHD cases in the school setting.

| Index |  | AUC | Sen | Spec | DOR |  |  |
| --- | --- | --- | --- | --- | --- | --- | --- |
| CBCL-IA | N = 125 | 0.889 | 0.82 | 0.75 | 13.67 |  |  |
|  | N = 122 | 0.884 | 0.81 | 0.75 | 12.79 |  |  |
| TRF-IA | N = 125 | 0.817 | 0.77 | 0.79 | 12.60 |  |  |
|  | N = 122 | 0.809 | 0.75 | 0.79 | 11.28 |  |  |
| YSR-IA | N = 125 | 0.726 | 0.68 | 0.68 | 4.52 |  |  |
|  | N = 122 | 0.725 | 0.67 | 0.68 | 4.31 |  |  |
| CPT-OE | N = 125 | 0.647 | 0.66 | 0.61 | 3.04 |  |  |
|  | N = 122 | 0.65 | 0.51 | 0.71 | 2.55 |  |  |
| QIK-OE | N = 125 | 0.643 | 0.66 | 0.59 | 2.79 |  |  |
|  | N = 122 | 0.646 | 0.66 | 0.59 | 2.79 |  |  |
| CPT-CE | N = 125 | 0.486 | 0.55 | 0.47 | 1.08 |  |  |
|  | N = 122 | 0.475 | 0.32 | 0.73 | 1.27 |  |  |
| QIK-CE | N = 125 | 0.441 | 0.39 | 0.53 | 0.72 |  |  |
|  | N = 122 | 0.441 | 0.43 | 0.48 | 0.70 |  |  |
| **Note.** AUC = area under the curve, Sen = sensitivity, Spec = specificity, DOR = Diagnostic Odds Ratio, IA = inattention, OE = omission errors, CE = comission errors. Sen & Spec belong to the depicted index-values. | | | | | | |  |
